# Zero‐Valent Nickel Stabilized in Surface‐Supported Metal–Organic Chains

**DOI:** 10.1002/anie.1508970

**Published:** 2026-07-24

**Authors:** Simone Mearini, Dominik Brandstetter, Mira S. Arndt, Olga Resel, Daniel Baranowski, Yan Yan Grisan Qiu, Peter Puschnig, Martin Sterrer, Giovanni Zamborlini, Vitaliy Feyer, Claus M. Schneider

**Affiliations:** ^1^ Peter Grünberg Institute (PGI‐6) Jülich Research Center Jülich Germany; ^2^ Institute of Physics, NAWI Graz University of Graz Graz Austria; ^3^ Department of Physics TU Dortmund University Dortmund Germany; ^4^ Present address: Physical and Computational Sciences Directorate and Institute for Integrated Catalysis Pacific Northwest National Laboratory Richland Washington USA; ^5^ Faculty of Physics and Center for Nanointegration Duisburg‐Essen (CENIDE) University of Duisburg‐Essen Duisburg Germany; ^6^ Department of Physics and Astronomy UC Davis Davis USA

**Keywords:** density functional theory, metal–organic chains, scanning tunnelling microscopy, transition metal, zero‐valent state

## Abstract

The stabilization of zero‐valent nickel Ni(0) centers in extended coordination networks remains a major challenge due to their pronounced reactivity and susceptibility to oxidation. Here, we demonstrate that linear coordination confinement within a surface‐supported metal–organic chain enables the stabilization of Ni(0) atoms on Ag(100). The symmetry of 9,10‐dicyanoanthracene (DCA), combined with substrate templating effects, directs the formation of an ordered linear Ni‐DCA coordination motif. Spectroscopic measurements provide unambiguous evidence for a formal Ni(0) oxidation state, representing a rare realization of a zero‐valent transition metal center within an extended, atomically defined system. The electronic structure is governed by hybridization between Ni 3d orbitals and the π‐system of DCA, resulting in an anisotropic electronic dispersion with predominantly one‐dimensional character. These results establish linear coordination as a strategy to stabilize reactive, low‐valent metal centers in surface‐supported metal–organic architectures.

## Graphical abstract

9,10‐Dicyanoanthracene coordinates Ni atoms on Ag(100) to form metal–organic chains that stabilize zero‐valent Ni(0) (d^10^) centers with quasi‐one‐dimensional metal–ligand electronic coupling.

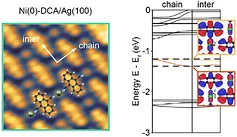

## 1 Introduction

The stabilization of zero‐valent transition‐metal (TM) centers in well‐defined coordination environments provides access to highly electron‐rich, often nucleophilic metal sites that underpin a wide range of applications in catalysis, molecular electronics, and materials design [1, 2, 3, 4]. In this context, nickel in its zero‐valent oxidation state, Ni(0), is particularly attractive owing to its high valence electron density and versatile redox chemistry. Indeed, isolated Ni(0) atoms, as well as transition metals in analogous electronic configurations such as Pd(0), have demonstrated remarkable versatility in chemical transformations, including cross‐coupling reactions [5, 6, 7, 8]. However, the same electron‐rich character that underlies this reactivity also renders Ni(0) highly susceptible to oxidation, thereby restricting its stabilization mainly to discrete molecular complexes synthesized in solution. Such complexes rely on ligands that enable the coexistence of ligand‐to‐metal σ‐donation and metal‐to‐ligand π‐backdonation, including carbon monoxides [1, 2], phosphines [9, 10, 11], cyanides [3], and N‐heterocyclic carbenes [12], thereby preserving the low‐valent oxidation state and suppressing oxidative degradation [11, 13]. Beyond molecular complexes, Ni(0) has also been identified in selected organometallic nanoparticles, where ligand‐field effects and the surrounding electronic environment inhibit oxidation [14, 15].

Extending the stabilization of Ni(0) to atomically precise, low‐dimensional materials remains a critical and largely unexplored challenge. On‐surface coordination chemistry provides a versatile platform for constructing extended metal–organic architectures in which metal centers interact with both the organic ligands and the underlying metallic substrate [16]. In most surface‐supported coordination networks reported to date, nickel adopts positive oxidation states (Ni(I), Ni(II)) due to charge transfer to the coordinating ligands [17, 18, 19, 20, 21]. In contrast, zero‐valent transition metals acting as structural nodes within surface‐confined metal–organic architectures are exceedingly rare [22], primarily due to their inherent reactivity and nucleophilic character. Nevertheless, isolated transition‐metal atoms stabilized in well‐defined surface‐supported coordination environments can exhibit remarkable chemical activity [23, 24]. For example, adsorbed Rh single atoms in linear coordination environments have been shown to participate actively in hydrogen activation processes relevant to catalytic hydrogenation [24].

These observations suggest that controlling the coordination geometry of surface‐supported metal centers may provide a route to stabilize reactive low‐valent states. In this context, surface‐supported linear metal–organic architectures represent a particularly promising platform. Their low coordination number and linear geometries intrinsically concentrate metal–ligand interactions along a single axis, potentially enhancing directional electronic coupling. In particular, the symmetry of 9,10‐dicyanoanthracene (DCA, D_2h_ point group) makes it a suitable ligand for guiding the growth of surface‐confined linear chains. When combined with substrate templating effects, the molecular geometry of DCA can favor extended linear metal–organic arrangements with well‐defined coordination environments.

Here, we report the synthesis of a surface‐confined metal–organic chain (MOC) composed of Ni(0) centers coordinated by DCA on Ag(100). By combining scanning tunnelling microscopy (STM), x‐ray photoelectron spectroscopy (XPS), momentum‐resolved valence band spectroscopy, and density functional theory (DFT), we provide a comprehensive structural and electronic characterization of this system. XPS and DFT confirm the formal Ni(0) oxidation state, while momentum‐resolved spectroscopy reveals hybrid states arising, which DFT simulations attribute to metal–organic interaction between Ni 3d orbitals and the DCA π‐system, exhibiting pronounced unidirectional dispersion. These results establish that the linear coordination confinement, together with directional ligand interactions, provides an effective strategy for stabilizing zero‐valent metals within extended surface‐supported architectures.

## 2 Results and Discussion

**FIGURE 1:**
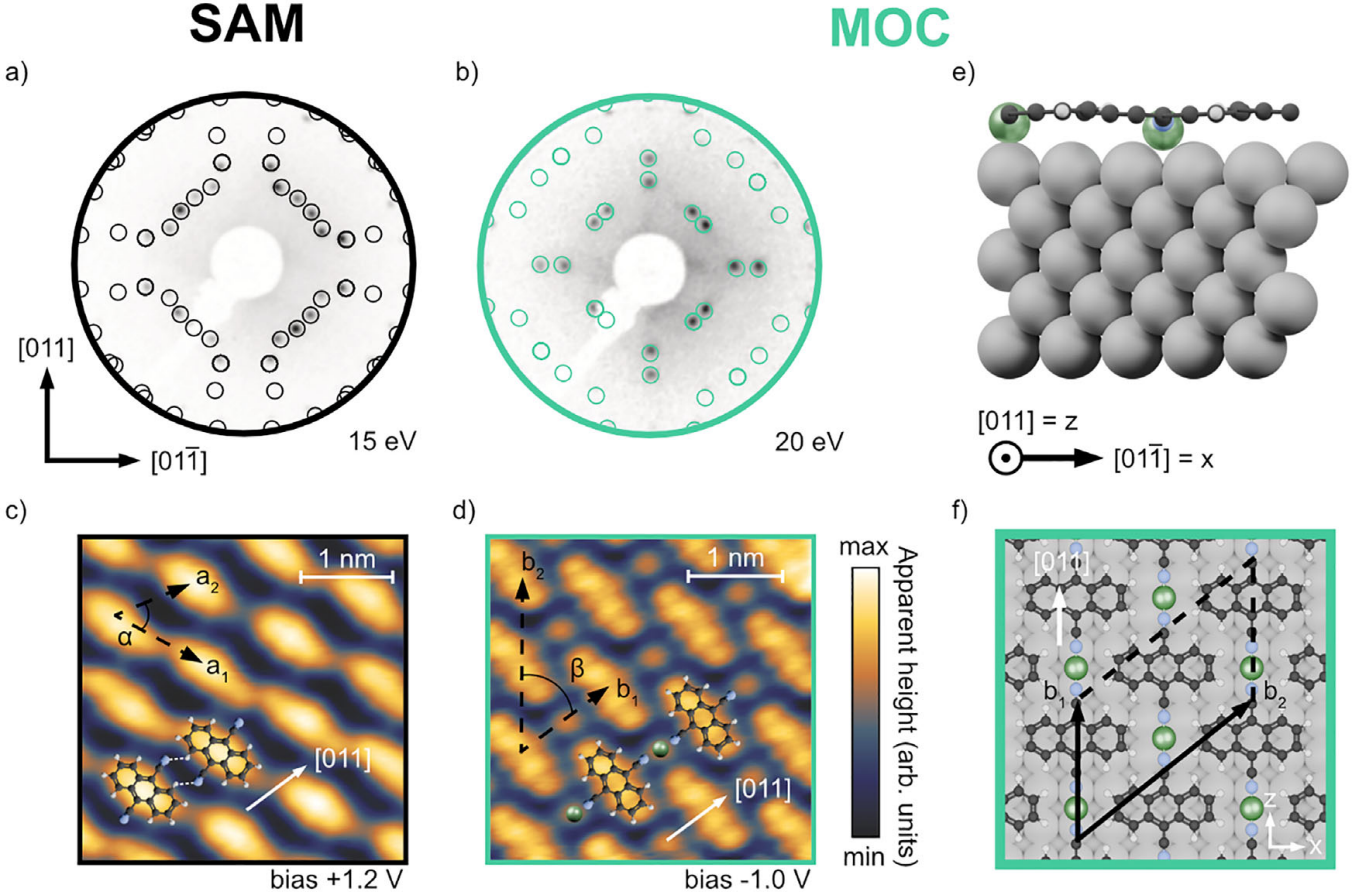
(a and b) LEED patterns and corresponding simulations (circles) acquired for (a) the SAM (black) at 15 eV and for (b) the MOC (teal) at 20 eV. (c and d) STM images acquired for (c) the SAM (image size 3.5 × 3.5 nm^2^, bias voltage +1.2 V, setpoint current 0.8 nA) and (d) the MOC (image size 3.4 × 3.4 nm^2^, bias voltage ‐1.0 V, setpoint current 0.8 nA). (e) Side view and (f) top view of the relaxed structural model of the MOC on five layers of Ag(100).

The deposition of DCA, whose molecular structure is shown in Figure S1, onto an Ag(100) surface results in the formation of a self‐assembled monolayer (SAM). The sharp features observed in the low‐energy electron diffraction (LEED) pattern presented in Figure 1a demonstrate the long‐range order of the network. Simulation of the LEED pattern (black overlay in Figure 1a) reveals an incommensurate epitaxy of ((3.7, 0.9), (1.3, 2.8)) with respect to the underlying Ag(100) surface. The corresponding monoclinic unit cell is characterized by the lattice parameters a_1_  =  11.0  ±  0.3 Å, a_2_  =  8.9  ±  0.3 Å, and an interaxial angle α  =  51  ±  1°, rotated by approximately 14  ±  1° relative to the substrate [011] high‐symmetry direction.

High‐resolution STM imaging (Figure 1c), acquired at a sample bias of +1.2 V, reveals a periodic stripe‐like molecular arrangement, with the cyano groups aligned nearly parallel to the [011] direction. Owing to its extended π‐conjugation, the anthracene backbone of DCA is expected to adopt a largely flat adsorption geometry on Ag(100), reflecting the rigidity of its fully aromatic framework [25, 26, 27]. Such a configuration limits the downward tilting of the cyano functional groups toward the surface and thereby weakens molecule–substrate interactions.

Consistent with these reduced substrate templating effects, the molecular layer adopts an incommensurate epitaxy, as determined from LEED. Additionally, the packing motif resolved in STM is compatible with the formation of intermolecular double C─H···N hydrogen bonds of ∼2.4 Å between adjacent molecules (dotted lines in Figure 1c), which likely contribute to stabilizing the stripe‐like arrangement within the SAM [28, 29, 30, 31, 32].

Upon incorporation of Ni atoms at a sample temperature of 370 K, the LEED pattern changes markedly while remaining well defined (Figure 1b). The corresponding simulation (teal overlay) identifies a commensurate epitaxy of ((5, 4), (0, 4)), yielding a new unit cell with lattice parameters b_1_ = 11.6  ±  0.3 Å, b_2_  =  18.5  ±  0.3 Å and an interaxial angle of β  =  51 ±  1°, with b_1_ aligned with the [011] direction of the substrate. STM images acquired after Ni deposition (Figure 1d) at a −1.0 V bias clearly resolve alternating DCA molecules, appearing as large oval features, and smaller circular protrusions attributed to the Ni sites. These features arrange in a staggered geometry that minimizes steric repulsion between adjacent anthracene backbones.

These findings suggest a predominantly unidirectional coordination pathway along the N─Ni─N direction, which coincides with the [011] direction in Figure 1d. Such a linear coordination motif is structurally enabled by the mirror‐symmetric arrangement of the two functional groups most directly involved in the metal–ligand interaction on the anthracene moiety in DCA. The formation of the linear coordination motif is further promoted by the symmetry of the Ag(100) substrate. Previous studies have shown that DCA‐based coordination assemblies on fcc(111) surfaces form hexagonal or Kagome‐like architectures, whereas the four‐fold symmetry of the fcc(100) surface favors unidirectional arrangements [33]. In the present case, the stripe‐like ordering already observed for the DCA‐SAM prior to Ni incorporation suggests that the substrate acts as a structural template for the subsequent formation of the Ni‐DCA chains. The resulting structure will, thus, hereafter be referred to as a metal–organic chain (MOC). The [011] direction along the coordination bonds will then be denoted as the “chain” direction, whereas the perpendicular $\left[01\bar{1}\right]$ will be defined as the “inter”‐chain direction.

Based on the experimental LEED pattern and the STM images we have constructed a structural model of the MOC (side and top view in Figure 1e, f, respectively), whose geometry is relaxed by density functional theory (DFT) calculations (details below). Within the unit cell, two non‐equivalent Ni adsorption sites are identified, namely hollow and bridge sites, resulting in a vertical displacement of approximately 0.7 Å and 0.4 Å below the molecular plane, respectively. The resulting Ni─N bond length of 1.75 Å is notably shorter than values reported for Ni‐cyano coordination in related surface‐supported metal–organic frameworks, indicating strong metal–ligand interaction [18, 19, 20, 21].

**FIGURE 2:**
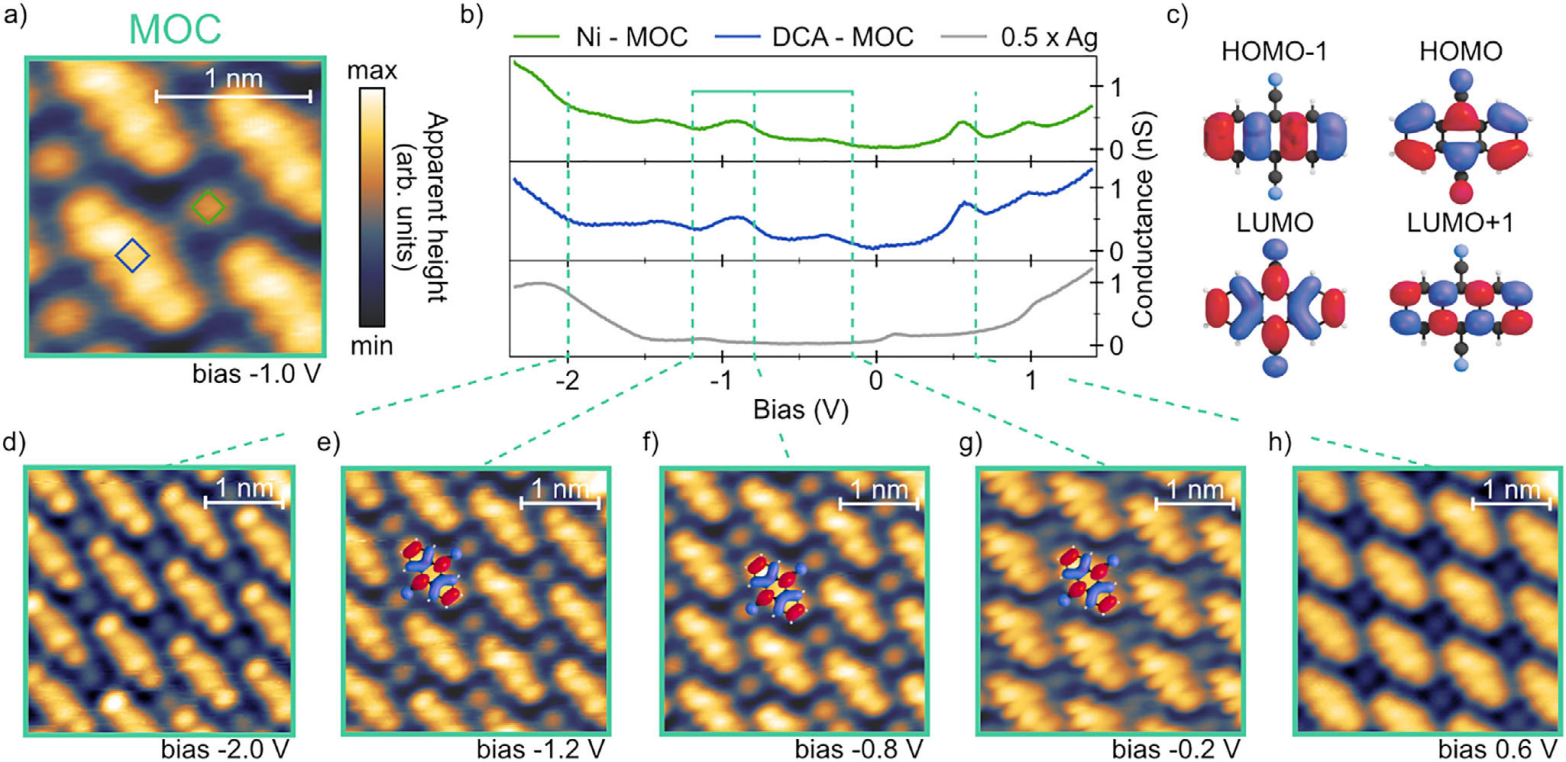
(a) STM image (image size, 1.9 × 1.9 nm^2^, bias voltage −1.0 V, setpoint current 0.8 nA) of the MOC; the blue and green diamond shapes represent the centers of a DCA molecule and a Ni atom, respectively. (b) STS spectra acquired, from top to bottom, for the Ni center (green), the DCA center (blue) and the Ag substrate (gray) of the MOC; the dashed lines indicate the main features present in occupied and unoccupied states. (c) Real‐space representations of the frontier molecular orbitals calculated for freestanding DCA. (d–g) STM images (image size 3.4 × 3.4 nm^2^, setpoint current 0.8 nA) at (d) −2.0 V, (e) −1.2 V, (f) −0.8 V, (g) −0.2 V, (h) +0.6 V bias voltage for the MOC.

Although STM and LEED measurements already suggest the presence of coordinative interactions between Ni and DCA, direct confirmation of metal–organic hybridization and its impact on the electronic properties of the network requires further spectroscopic evidence. Initial insight into this behavior is provided by STS measurements acquired at the Ni center (green) and at the center of the DCA molecules (blue) within the MOC (Figure 2b), as indicated in the close‐up STM image in Figure 2a. For comparison, an STS spectrum obtained on the bare Ag surface (gray) is also shown, with the probed area reported in Figure S2.

Distinct spectral features at −1.2, −0.8, and −0.2 V are clearly observed in the STS curves recorded for both Ni centers and DCA molecules, while being absent on the bare Ag surface. This indicates that these states are associated with the metal–organic chain and are electronically delocalized across both Ni and DCA moieties. Further insight is gained by comparing bias‐dependent STM images of the MOC (Figure 2d–h) with the calculated Kohn–Sham orbitals of free‐standing DCA (Figure 2c). In particular, the spatial shape of the lowest unoccupied molecular orbital (LUMO), characterized by five nodal planes along the anthracene backbone, can be clearly identified at all the bias voltages between −1.2 V and −0.2 V (Figure 2e–g). These observations suggest at least partial occupation of the DCA LUMO.

At more negative (−2.0 V) and positive (+0.6 V) bias voltages (Figure 2d, h), the nodal structure of DCA becomes less recognizable, rendering the molecular orbital assignment ambiguous. Additionally, the contrast at the Ni sites is significantly reduced, indicating weaker Ni contributions and a predominant charge localization on the molecular backbone.

**FIGURE 3:**
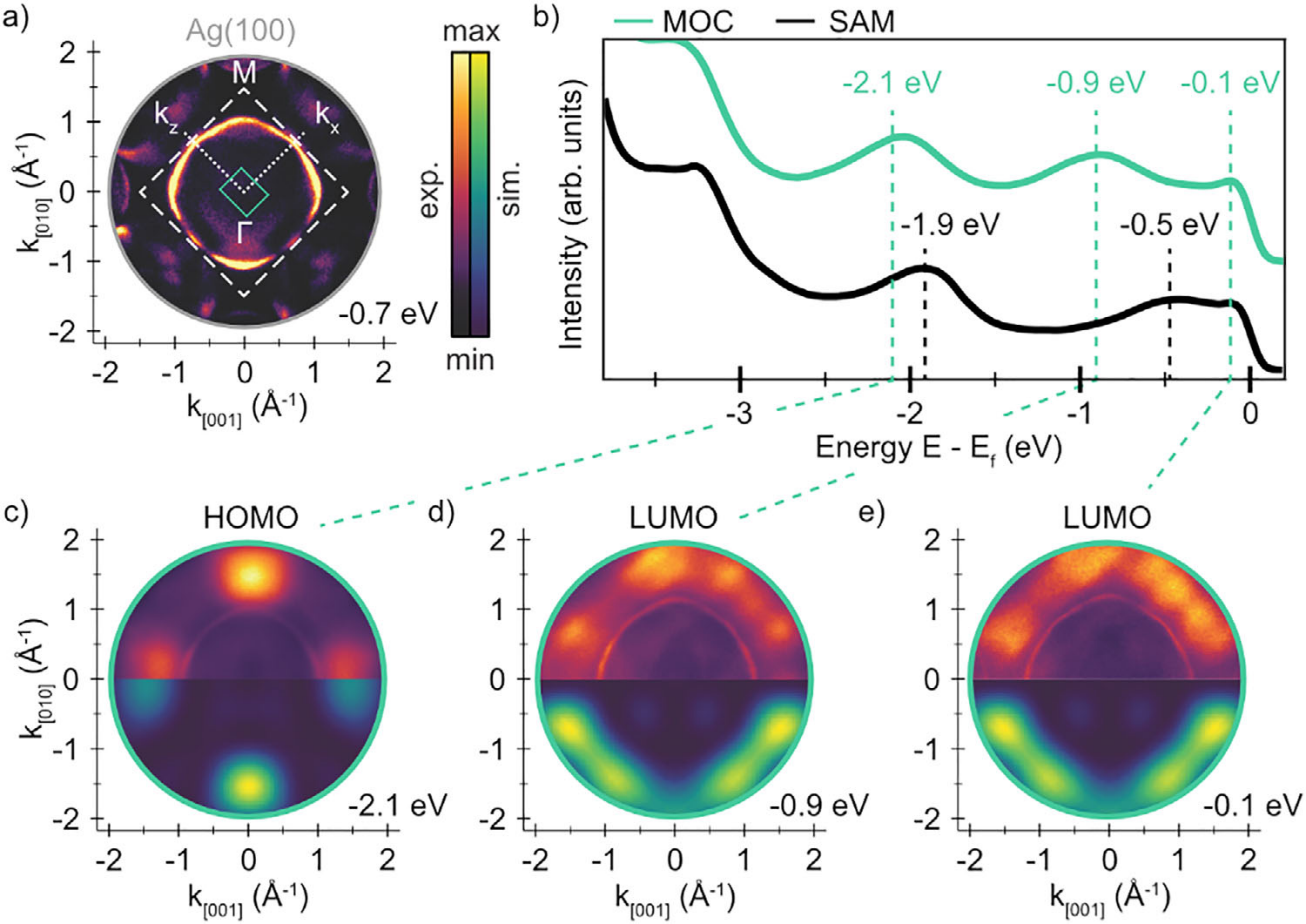
(a) Angle‐resolved photoemission momentum map of the reference Ag(100) surface at −0.7 eV energy. The dotted lines indicate the chain and inter‐chain directions, indicated by Γ  −  *k_z_* and Γ  −  *k_x_*, respectively; the teal parallelogram represents the first Brillouin zone of the MOC. (b) Valence band spectrum (*h*ν = 30 eV, p‐polarized light) of the SAM (black) and MOC (teal). (c–e) Experimental (top, *h*ν = 30 eV, p‐polarized light) and simulated (bottom, supported model) momentum maps of the MOC system at (c) −2.1 eV, (d) −0.9 eV, and (e) −0.1 eV energy.

To further substantiate this orbital assignment, the electronic structure has been directly probed using a photoemission electron microscope (PEEM), operating in momentum‐resolved mode. The experiments were performed using p‐polarized light at a photon energy of 30 eV and a sample temperature of 90 K. These measurements yield three‐dimensional stacks made of momentum maps (*k*_[001]_, *k*_[010]_) as a function of the energy. Integration of the full momentum distribution at each energy slice produces the valence band (VB) spectra shown in Figure 3b for the pristine SAM (black curve) and the Ni‐DCA MOC (teal curve), and in Figure S3 for the bare Ag(100) reference (gray curve). In the following discussion, we adopt the unconventional representation of the VB energy as *E* − *E_f_* to enable direct comparison with the STS data.

Upon Ni incorporation, the SAM feature centered at −1.9 eV shifts by approximately −0.2 eV, giving rise to the peak at −2.1 eV in the MOC spectrum. In contrast, the SAM feature at −0.5 eV does not seem to have a direct counterpart in the MOC. Instead, two new features emerge at −0.9 and −0.1 eV.

To elucidate the nature of these states, the experimental momentum maps at the corresponding energies were extracted for both the SAM (Figure S4) and the MOC (Figure 3c–e, top half) and directly compared with simulated momentum maps calculated for a gas phase DCA molecule [34]. This comparison allows for an unambiguous assignment of the spectral features. The peaks at −1.9 eV (SAM) and −2.1 eV (MOC) originate from the highest occupied molecular orbital (HOMO) (Figures 3c, S4a), whereas the features at −0.5 eV for the SAM and at −0.9 and −0.1 eV for the MOC are attributed to LUMO‐derived states (Figures 3d, e, S4b). These results confirm that partial occupation of the LUMO already occurs in the SAM due to charge transfer from the substrate to the molecular layer. Moreover, the appearance of multiple LUMO‐derived features in the MOC corroborates the observation of LUMO splitting in multiple components upon Ni coordination, as evidenced in the bias‐dependent STM images (Figure 2e–g).

Such splitting is characteristic for the formation of bonding and antibonding states [35, 36], with the bonding states shifting towards lower energies and the anti‐bonding states approaching the Fermi level.

While the experimental results establish the existence of strong Ni–DCA interactions, density functional theory calculations are required to fully rationalize the nature of the hybridization in this unique coordination environment. The relaxed structural model of the supported MOC is shown in Figure 1e, f and includes five layers of Ag substrate, with the bottom three layers frozen in the bulk geometry. The calculations were performed using VASP with the PBE exchange‐correlation functional and a self‐interaction error correction in the GGA+U Dudarev ansatz (U = 3 eV) applied to the Ni states. For details on the simulation, we refer to the Methods section (Supporting Information).

**FIGURE 4:**
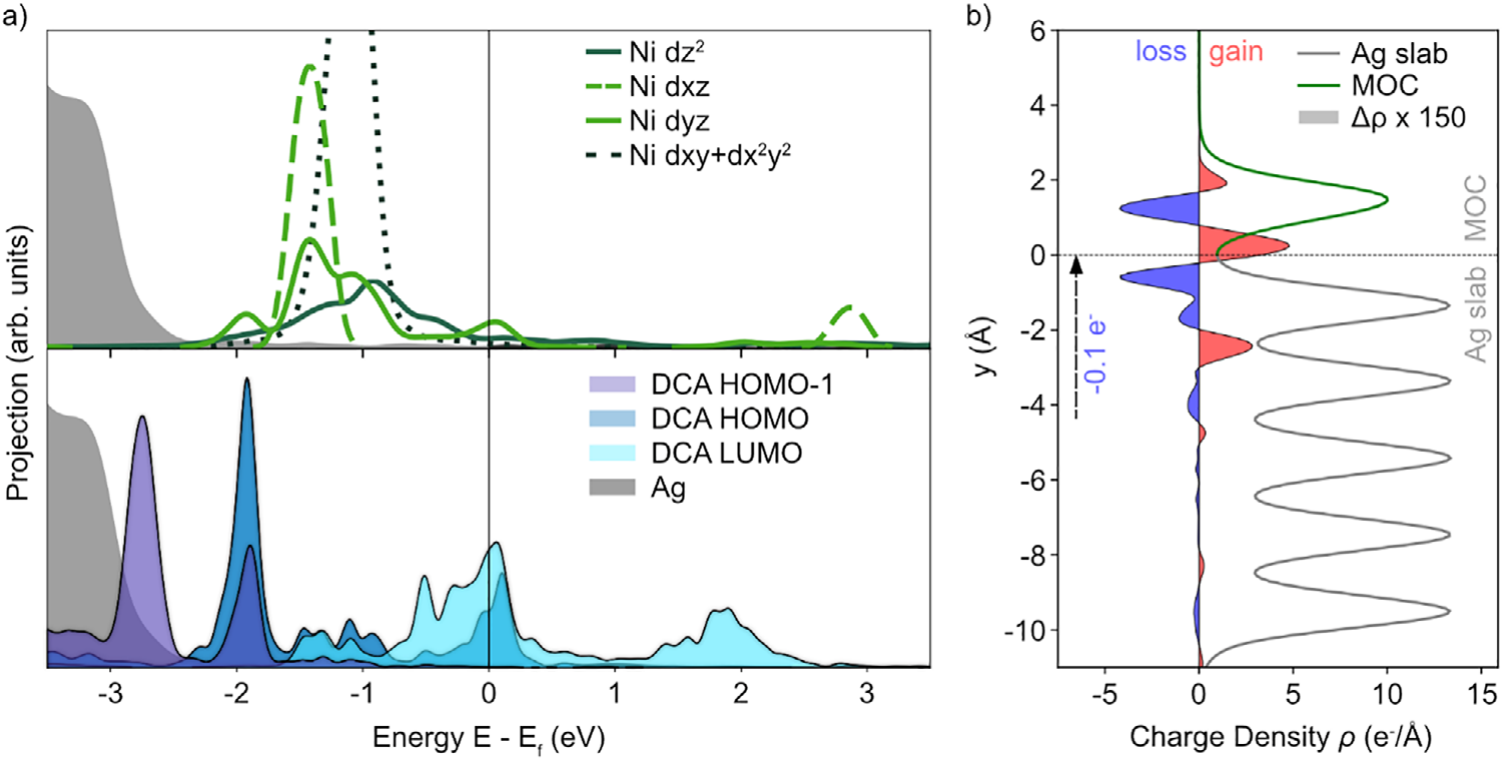
(a) Density of states projected onto Ni 3d states (top panel) and DCA frontier orbitals (bottom panel) for the relaxed structural model of the supported MOC; (b) Plane‐averaged total charge density ρ (lines), and charge density difference Δρ (shaded area; scaled by a factor of 150) as a function of the height y parallel to the interface normal for the supported MOC. The red and blue areas indicate regions with an overall gain or loss in electrons compared to the bare substrate / freestanding MOC layer, respectively.

The projected density of states (PDOS) for the MOC is shown in Figure 4a, with the Ni 3d contributions (green curves) and molecular states (shaded areas) displayed in the upper and lower panels, respectively. Even though Ni 3d states show some non‐vanishing density of states above the Fermi edge (larger energy range shown in the SI, Figure S5), integration of the Ni 3d projection yields a formal 3d^10^ electron configuration, corresponding to a Ni(0) oxidation state. Furthermore, spin unrestricted calculations converge toward a spin‐unpolarized solution with no local magnetic moment on the Ni centers, indicating a diamagnetic ground state of the supported chains. This assignment is independently corroborated by XPS data at the Ni 2p_3/2_ core‐level (Figure S6a), where the main peak at 852.2 eV coincides with the binding energy of Ni(0) and is clearly distinct from the typical positions associated with Ni(I) and Ni(II) species reported for related metal–organic arrays [17, 18, 37]. For comparison, Figure S7 shows the XPS spectrum of the Ni 2p_3/2_ core‐level region for a Ni reference prepared by depositing an excess of Ni directly onto Ag(100). Additional evidence for coordination‐induced electronic redistribution is provided by the N 1s and C 1s core‐level spectra shown in Figure S6. Upon formation of the Ni‐DCA chains, the N 1s feature shifts by approximately +0.3 eV toward higher binding energy, while the nitrile‐related shoulder in the C 1s spectrum shifts toward lower binding energies by a comparable amount. These opposite shifts within the cyano group are consistent with charge redistribution associated with the Ni–DCA coordination interaction.

With the oxidation state firmly established, the orbital‐resolved PDOS in Figure 4a is examined to elucidate the nature of the metal–ligand hybridization. The local coordinate system is chosen such that the *z*‐axis is aligned along the N─Ni─N coordination axis (chain direction), the *x*‐axis lies along the inter‐chain direction, and the *y*‐axis is oriented perpendicular to the surface (see Figure 1f). Within this framework, the *d_xy_* and $d_{x^{2}y^{2}}$ orbitals are grouped together for clarity, appearing as a single sharp feature. This behavior is easily understood within the crystal field picture for a linear D_∞h_ coordination environment, where five‐fold degenerate Ni 3d orbitals split into discrete energy levels, including a doubly degenerate {$d_{xy},d_{x^{2}-y^{2}}$} set. Owing to their radial orientation perpendicular to the Ni─N bond, these orbitals lack the appropriate symmetry for the overlap with the π‐character frontier molecular orbitals of DCA and therefore remain largely unaffected by the coordination.

In contrast, the nominally degenerate {*d_xz_*, *d_yz_*} orbitals exhibit a markedly different behavior. Specifically, the *d_xz_* appears as a largely localized peak at −1.5 eV and a smaller contribution at +2.9 eV, whereas the *d_yz_* has a strongly dispersive broad peak in the energy range of −2.1 to +0.1 eV. This apparent deviation from a simple crystal‐field description reflects the limitation of the point charge approximation and highlights the importance of orbital symmetry matching. The frontier molecular orbitals of DCA predominantly possess π‐character, which matches the symmetry of the *d_yz_* orbital in the chosen local coordinate system. This enables hybridization with both the HOMO and LUMO of DCA and provides microscopic explanation for the experimentally observed splitting of the LUMO into bonding and antibonding states.

A schematic representation of the dominant orbital interactions within the Ni‐DCA chains is shown in Figure S8a, highlighting the π‐type hybridization between the Ni *d_yz_* orbital and the DCA frontier orbitals. Although the *d_xz_* orbital possesses in‐plane π‐symmetry, with its nodal plane oriented perpendicular to the molecular plane, this symmetry is less common among the frontier molecular states of DCA. An exception is provided by higher‐lying LUMO+x orbitals where hybridization can take place, as evidenced by the second peak located at +2.9 eV in the PDOS (see Figure S8c).

Finally, the $d_{z^{2}}$ orbital exhibits a very broad spectral projection, predominantly located in the valence region between −2 eV and the Fermi level, but extending far into the conduction band up to +6 eV. Additional features are observed even below the Ag d‐band (Figure S5). With the *z*‐axis aligned parallel to the bonding direction, these peaks at low energies can be rationalized as σ‐symmetric bonding states involving deeper‐lying molecular orbitals, schematically illustrated in Figure S8b. However, the limited energetic overlap with these states alone cannot explain the broad spectral distribution of the $d_{z^{2}}$ contribution in the PDOS (Figure 4a). These observations point to a significant contribution from direct hybridization between the Ni $d_{z^{2}}$ orbital and substrate‐derived states, consistent with re‐localization in energy of the $d_{z^{2}}$ observed upon removal of the Ag(100) substrate (Figure S9).

Charge density difference calculations were performed for the supported MOC (Figure 4b). These reveal a small charge redistribution at the interface, characterized by charge depletion within the topmost Ag layer and charge accumulation within the metal–organic layer. Integration of the charge density difference (*Δρ*) yields a total charge transfer of approximately 0.105 *e* ^−^ from the substrate to the MOC. Although comparatively small, this charge redistribution confirms the presence of weak adsorbate–substrate coupling and supports the role of the Ag(100) surface in stabilizing the supported Ni–DCA architecture. At the same time, the limited magnitude of the charge transfer indicates that the substrate contribution remains secondary to the dominant Ni–DCA coordination interaction.

To isolate the contribution of the Ag(100) substrate, calculations were repeated after removing the substrate from the model, while keeping the MOC geometry frozen (Figure S9, top half). In this substrate‐free configuration, the delocalization of the $d_{z^{2}}$ state is strongly reduced, confirming that adsorbate–substrate coupling plays an important role in shaping its electronic structure.

A residual splitting of the $d_{z^{2}}$ feature persists in this geometry, which can be attributed to partial hybridization with DCA orbitals enabled by the vertical displacement of the Ni atoms (0.4 Å–0.7 Å) below the molecular plane, resulting in symmetry breaking. When the MOC is fully relaxed in a substrate‐free configuration (Figure S9, bottom half), the structure becomes planar, the symmetry is restored, and the splitting collapses into a single sharp $d_{z^{2}}$ peak. This establishes that both symmetry breaking and substrate interaction are essential for the observed hybridization of the $d_{z^{2}}$ orbital.

**FIGURE 5:**
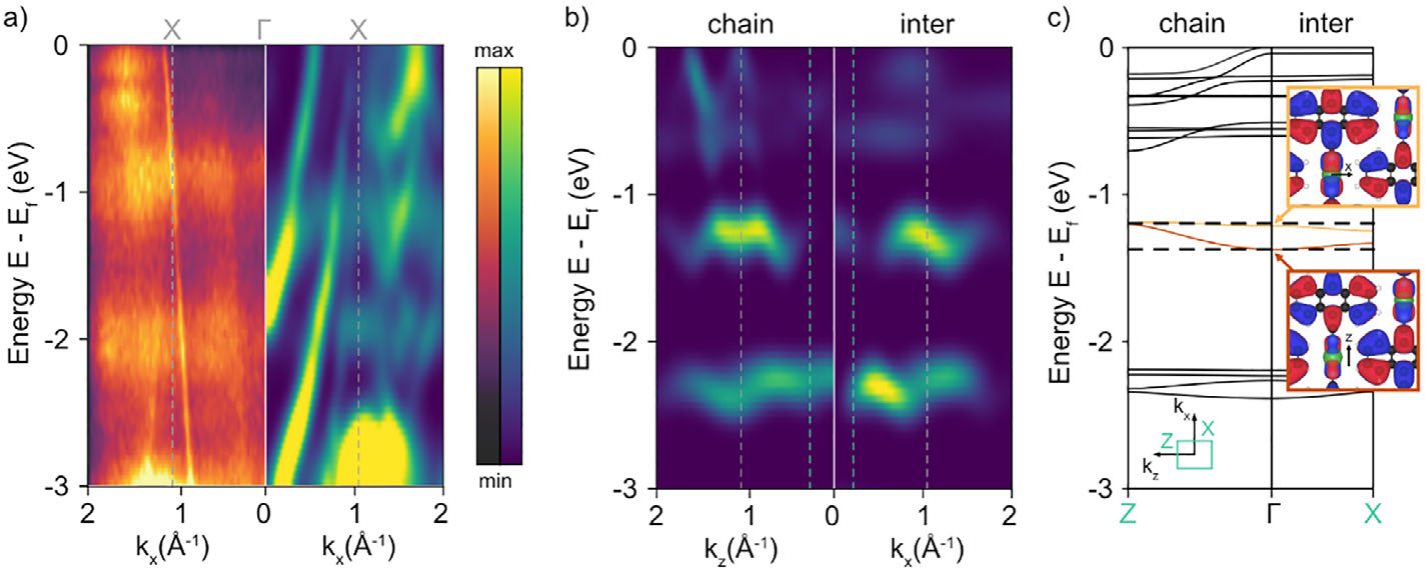
(a) Experimental (left‐half, *h*ν = 30 eV, p‐polarized light) vs. simulated (right‐half, supported model) band maps of the MOC along the substrate Γ—Χ directions. (b) Simulated band map of the substrate‐free frozen MOC along the *k_x_* (inter‐chain)/*k_z_* (chain) direction in reciprocal space ranging from the Γ‐point to the edge of first Brillouin zone of the MOC, respectively. The gray and teal dashed lines indicate the edges of the first Brillouin zone of Ag(100) and the MOC, respectively. (c) Simulated photoemission quasi‐band structure for the substrate‐free frozen MOC along the Γ—Χ (inter‐chain) and Γ—Ζ (chain) directions. The Ζ and Χ labels highlighted in teal indicate the edges of the first Brillouin zone of the MOC along the chain and inter‐chains directions, respectively. The simulation data shown was purposefully not symmetrized to represent only a single growth domain. The insets show the real space representation of the Bloch states at the Γ point for the bonding and antibonding combination of neighboring chains, highlighted in yellow and orange, respectively.

Lastly, we further investigate the predominantly unidirectional character of the Ni–DCA coordinative interactions with photoemission band maps. Owing to the chain‐like arrangement of the Ni‐DCA network, stronger electronic coupling is expected along the chain direction than along the inter‐chain direction. Experimentally, however, this anisotropy cannot be resolved due to the presence of multiple mirror domains aligned with the high symmetry directions of the substrate (Figure S10). Specifically, cutting the 3D stack along the substrate Γ − X direction results in the band map shown in Figure 5a (left half), where chain and inter‐chain features are overlapped. The close resemblance to the symmetrized band map simulated for the supported MOC (Figure 5a, right half) confirms the overlap of contributions from multiple domains and the impossibility to discern between the two directions.

To overcome this limitation, Figure 5b presents the simulated photoemission band map of the substrate‐free frozen MOC model (Figure S9, top half), where the left and right halves correspond to reciprocal‐space cuts along the chain (*k_z_*) and inter‐chain (*k_x_*) directions, respectively. Removing the substrate allows the intrinsic MOC interaction to be isolated. However, charge transfer from the substrate is absent and, consequently, no significant LUMO occupation is expected (Figure S9, top half).

The analysis therefore focuses on the bright HOMO‐*d_yz_* derived hybrid states, which appear in the energy range between −1 eV and −1.6 eV and are shifted closer to the Fermi level compared to the experiment. These states exhibit an apparent dispersion of ∼0.5 eV along both chain and inter‐chain directions, seemingly suggesting a significant inter‐chain dispersion. The corresponding band structure (Figure 5b), however, reveals that this behavior originates from two nearly overlapping bands in this energy range, highlighted in yellow and orange. The corresponding real space representations of the Bloch states at the Γ‐point (insets in Figure 5c) reveal that these bands unambiguously correspond to the in‐phase and out‐of‐phase combinations of the two neighboring chains within the unit cell.

Along the inter‐chain direction, the energy separation of these two contributions gives rise to the appearance of a broad dispersive feature in the photoemission spectrum, even though the intrinsic dispersion of each band is limited to ∼40 meV. In contrast, along the chain direction, both bands exhibit genuine band dispersion, with the out‐of‐phase band (orange) reaching ∼170 meV, highlighting the quasi‐one‐dimensional character of the system [38].

Taken together, these results demonstrate that electronic coupling in the Ni–DCA system is predominantly confined to the chain direction, confirming the formation of a linear metal‐organic assembly with strongly anisotropic electronic properties.

## 3 Conclusions

This study establishes a strategy for stabilizing zero‐valent nickel coordinated in a surface‐supported linear metal–organic architecture via an interplay of ligand symmetry, substrate templating effects and electronic interactions. On Ag(100), DCA molecules self‐assemble into quasi‐linear arrangements that act as pre‐organized scaffold for subsequent Ni incorporation, enabling the formation of extended DCA–Ni–DCA coordination chains. Importantly, a comprehensive experimental and theoretical analysis of the chains identifies Ni sites stabilized in a formal Ni(0) oxidation state with a diamagnetic ground state.

Scanning tunnelling spectroscopy and momentum‐resolved valence band measurements demonstrate that Ni incorporation is responsible for the emergence of hybrid electronic states. Density functional theory elucidates their origin in selective hybridization between Ni 3d orbitals and the π‐system of DCA. Furthermore, the analysis of the calculated band structure shows that significant dispersion is confined along the chain direction, with only weak interactions between neighboring chains splitting the in‐phase and out‐of‐phase combinations, giving rise to an apparent dispersion in the photoemission band maps.

Overall, this work demonstrates that linear coordination confinement at a metallic surface provides an effective route to stabilize electron‐rich Ni(0) centers within extended metal–organic architectures, giving rise to strongly anisotropic electronic properties that emerge from directional metal–ligand interactions.

## Author Contributions

**Simone Mearini**: investigation, validation, formal analysis, data curation, writing – original draft, writing – review and editing, visualization. **Dominik Brandstetter**: writing – original draft, methodology, formal analysis, data curation, investigation, writing – review and editing, software. **Mira S. Arndt**: investigation, writing – review and editing, formal analysis. **Olga Resel**: investigation, writing – review and editing. **Daniel Baranowski**: investigation, writing – review and editing. **Yan Yan Grisan Qiu**: investigation, writing – review and editing. **Peter Puschnig**: methodology, supervision, validation, writing – review and editing, software, conceptualization, investigation, funding acquisition. **Martin Sterrer**: formal analysis, investigation, writing – review and editing, data curation. **Giovanni Zamborlini**: conceptualization, funding acquisition, supervision, writing – review and editing, methodology. **Vitaliy Feyer**: conceptualization, investigation, validation, methodology, project administration, supervision, funding acquisition, writing – original draft, writing – review and editing. **Claus M. Schneider**: supervision, funding acquisition, writing – review and editing, resources.

## Conflicts of Interest

The authors declare no conflicts of interest.

## Supporting information



The authors have cited additional References within the Supporting Information [39, 40, 41, 42, 43, 44, 45, 46, 47, 48, 49].**Supporting File**: anie73691‐sup‐0001‐SuppMat.docx.

## Data Availability

The data that support the findings of this study are available from the corresponding author upon reasonable request.
