# Supplementary material for: Zero‐Valent Nickel Stabilized in Surface‐Supported Metal–Organic Chains
Source: Angew Chem Int Ed Engl. 2026 Jul 24;65(39):e1508970. doi: 10.1002/anie.1508970 (PMC13592301; doi:10.1002/anie.1508970)
Supplement: Supplementary file 1 [file ANIE-65-e1508970-s001.docx]

Supporting Information

**Zero-valent Nickel Stabilized in Surface-supported Metal-organic Chains**

Simone Mearini,*^[a]^ Dominik Brandstetter,*^[b]^ Mira S. Arndt,*^[c]^ Olga Resel,^[b]^ Daniel Baranowski,^[a,d]^ Yan Yan Grisan Qiu,^[a]^ Peter Puschnig,^[b]^ Martin Sterrer,^[b]^ Giovanni Zamborlini,*^[b,c]^ Vitaliy Feyer,*^[a,e]^ and Claus M. Schneider.^[a,e,f]^

^a^ Peter Grünberg Institute (PGI-6), Jülich Research Centre, 52428 Jülich, Germany

^b^ Institute of Physics, NAWI Graz, University of Graz, 8010 Graz, Austria

^c^ Department of Physics, TU Dortmund University, 44227 Dortmund, Germany

^d^ Present address: Physical and Computational Sciences Directorate and Institute for Integrated Catalysis, Pacific Northwest National Laboratory, Richland, Washington 99354, USA

^e^ Faculty of Physics and Center for Nanointegration Duisburg-Essen (CENIDE), University of Duisburg-Essen, 47048 Duisburg, Germany

^f^ Department of Physics and Astronomy, UC Davis, Davis, CA 95616, USA

**Table of Contents**

Methods Section	3

Figure S1: Chemical structure of DCA	4

Figure S2: STM image acquired in a molecule-free region of the Ni-MOC (image size 6.8 × 6.8 nm2, bias voltage +1 V, setpoint current 0.05 nA) for the Ag(100) reference in the STS spectra.	4

Figure S3: Valence band spectrum ($\boldsymbol{h\nu}$ = 30 eV, p-polarized light) of the clean Ag(100) substrate.	5

Figure S4: Experimental (top, $\boldsymbol{h\nu}$ = 30 eV, p-polarized light) and simulated (bottom, free-standing DCA model) constant energy 2D momentum maps of the DCA-SAM system at a) -1.9 eV (HOMO) and b) -0.4 eV (LUMO).	5

Figure S5 a) Density of states projected onto Ni 3d states (top panel) and DCA frontier orbitals (bottom panel) for the relaxed structural model of the supported MOC in an extended energy range.	5

Figure S6: a) Ni 2p_3/2_ XPS spectrum (dots) of the MOC; the “Ni(0)”, “Ni(I)”, and ”Ni(II)” labels indicate the binding energy position of Ni atoms stabilized in different oxidation states. b) C 1s and c) N 1s XPS spectra (dots) of the MOC (teal), SAM (black), and clean Ag(100) substrate (grey).	6

Figure S7: Ni 2p_3/2_ XPS spectrum (dots of a Ni reference on Ag(100). The “Ni(0)” label indicates the binding energy position of Ni atoms stabilized in the corresponding oxidation state.	6

Figure S8: Schematic representations of the dominant orbital interactions within the Ni-DCA chains, associated with the
a) $\boldsymbol{d}_{\boldsymbol{yz}}$, b) $\boldsymbol{d}_{\boldsymbol{z}^{\mathbf{2}}}$, and c) $\boldsymbol{d}_{\boldsymbol{xz}}$ Ni states.	7

Figure S9: Density of states projected onto Ni 3d states (curves) and DCA frontier orbitals (shaded areas) for the substrate-free MOC with frozen geometry (top) and the MOC fully stabilized in a substrate-free configuration (bottom).	7

Figure S10: STM images acquired for the Ni-MOC (image size 3.4 × 3.4 nm^2^, bias voltage +1 V, set point current 0.8 nA) for the two rotational domains extending along the a) $[\mathbf{01}\bar{\mathbf{1}}]$ and b) $[\mathbf{011}]$ propagation directions.	8

**Methods Section**

**Sample preparation**

Standard cleaning procedures consisting of repeated cycles ionized argon (Ar^+^) sputtering and heating to 800 K were applied to the Ag(100) crystal. DCA was evaporated directly onto the substrate held at room temperature from a Knudsen cell heated up to 390 K. In the next step, nickel was deposited using an e-beam evaporator (flux 10 nA) onto the SAM while keeping the sample at 370 K to facilitate structural rearrangement. After each step, the surface structure was monitored by LEED to confirm the formation of both the SAM and the MOC.

**STM and STS measurements**

The experiments were performed at the University of Graz (Austria) using a commercial low-temperature STM system (CreaTec GmbH). Electrochemically etched tungsten (W) tips were used for imaging. The sample temperature was maintained at 77 K (liquid nitrogen temperature) during the measurements. STS measurements were conducted with a modulation voltage of 25 mV and an oscillation frequency of 2.35 kHz. The topographic images reported in Figure 2 were recorded at bias voltages corresponding not to the maxima of the STS peaks, but to the rising edge of the corresponding spectral features.

**Momentum-resolved photoemission measurements**

Valence band photoemission spectra, momentum maps, and band maps were obtained using a photoelectron emission microscope (PEEM) available at the NanoESCA beamline of the Elettra synchrotron in Trieste, Italy.^39,40^ P-polarized light (photon energy 30 eV) was used to irradiate the sample, which was continuously rastered during acquisition to reduce radiation damage.

The measurements were performed at a pressure below 1×10^-10^ mbar with the sample cooled to 90 K using an open-cycle cryostat. The total energy and momentum resolution of the PEEM were set to 100 meV and ± 0.05 Å^-1^, respectively.

**XPS measurements**

XPS measurements were performed using a Phoibos 100 hemispherical energy analyser (pass energy = 10 eV), yielding an overall energy resolution of 0.6 eV, and a Mg $K_{\alpha}$ X-ray source (h𝜈 = 1254.6 eV) with an intrinsic line width of approximately 0.7 eV, both from SPECS GmbH and available at the University of Graz (Austria).

The experiment was conducted in normal emission geometry with an X-ray incidence angle of 55° relative to the surface normal. The sample was exposed to room temperature under a base pressure below 2x10^-10^ mbar. The Ag 3d_5/2_ peak, set to a binding energy of 368.2 eV,^41^ was used as an energy reference.

**Theoretical methods**

We performed ground state density functional theory (DFT) calculations using the Vienna Ab-initio Simulation Package (VASP), version 5.4.5 on the Vienna Scientific Cluster 4 (VSC-4).^44,45^ The exchange-correlation effects were treated using the Perdew-Burke-Enzerdorf generalized gradient approximation (PBE-GGA)^46^, along with a Grimme D3 vdW-correction with Becke-Johnson damping.^47^ Within this GGA-type exchange-correlation functional, the inclusion of a self-interaction error correction for the strongly localized d-orbitals in the transition metals is crucial for an accurate representation of the hybridization observed in the experiments. Therefore, we introduced an effective Hubbard-U parameter of 3 eV using the Dudarev ansatz.^48^ All calculations were performed initially in a spin unrestricted formalism to allow the system to relax into the energetically most favourable spin configuration. After finding this state to be closed shell we proceeded with spin unpolarized calculations.

Starting with the experimentally determined structures, we fully relaxed all systems until all atomic forces were below 0.01 eV/Å. We modelled the interface in the repeated slab approach with at least 30 Å vacuum layer, adding a dipole layer within the vacuum region to address the electric field discrepancy between either side of the slab.^49^ The bulk of the silver substrate was modelled with a total of five layers, with relaxation permitted only in the top two layers during geometry optimization. The first Brillouin zone was sampled using a Γ-centred 7x5x1 grid. For simulating the photoelectron distribution, the photoemission process was approximated as a one-step process, where the final state was treated as a plane wave. Additionally, we included a damping of the substrate emissions according to Ref.^34^ of γ = 0.75 Å^-1^.

**Supporting Data**

**Figure S1**. Chemical structure of DCA.

Figure S2. STM image acquired in a molecule-free region of the Ni-MOC (image size 6.8 × 6.8 nm^2^, bias voltage +1 V, setpoint current 0.05 nA) for the Ag(100) reference in the STS spectra.

Figure S3. Valence band spectrum ($\boldsymbol{h\nu}$ = 30 eV, p-polarized light) of the clean Ag(100) substrate.

Figure S4. Experimental (top, $\boldsymbol{h\nu}$ = 30 eV, p-polarized light) and simulated (bottom, free-standing DCA model) constant energy 2D momentum maps of the DCA-SAM system at a) -1.9 eV (HOMO) and b) -0.4 eV (LUMO).

Figure S5. a) Density of states projected onto Ni 3d states (top panel) and DCA frontier orbitals (bottom panel) for the relaxed structural model of the supported MOC in an extended energy range.

Figure S6. a) Ni 2p_3/2_ XPS spectrum (dots) of the MOC; the “Ni(0)”, “Ni(I)”, and ”Ni(II)” labels indicate the binding energy position of Ni atoms stabilized in different oxidation states. b) C 1s and c) N 1s XPS spectra (dots) of the MOC (teal),
SAM (black), and clean Ag(100) substrate (grey).

Figure S7. Ni 2p_3/2_ XPS spectrum (dots) of a Ni reference on Ag(100). The “Ni(0)” label indicates the binding energy position of Ni atoms stabilized in the corresponding oxidation state.

Figure S8. Schematic representations of the dominant orbital interactions within the Ni-DCA chains,
associated with the a) $\boldsymbol{d}_{\boldsymbol{yz}}$, b) $\boldsymbol{d}_{\boldsymbol{z}^{\boldsymbol{2}}}$, and c) $\boldsymbol{d}_{\boldsymbol{xz}}$ Ni states.

Figure S9. Density of states projected onto Ni 3d states (curves) and DCA frontier orbitals (shaded areas) for the substrate-free MOC with frozen geometry (top) and the MOC fully stabilized in a substrate-free configuration (bottom).

Figure S10. STM images acquired for the Ni-MOC (image size 3.4 × 3.4 nm^2^, bias voltage +1 V, set point current 0.8 nA) for the two rotational domains extending along the a) $\boldsymbol{[01}\bar{\boldsymbol{1}}\boldsymbol{]}$ and b) $\boldsymbol{[011]}$ propagation directions.
